# A cold high-pressure system over North China hinders the southward migration of *Mythimna separata* in autumn

**DOI:** 10.1186/s40462-022-00360-3

**Published:** 2022-12-01

**Authors:** Jian Zhu, Xiao Chen, Jie Liu, Yuying Jiang, Fajun Chen, Jiahao Lu, Hui Chen, Baoping Zhai, Don R. Reynolds, Jason W. Chapman, Gao Hu

**Affiliations:** 1 Department of Entomology, Nanjing Agricultural University, 1 Weigang Road, Nanjing, 210095 China; 2 State Key Laboratory of Biological Interactions and Crop Health, Nanjing Agricultural University, Nanjing, 210095 China; 3 College of Life Science, International Cooperative Research Centre for Cross-Border Pest Management in Central Asia, Xinjiang Normal University, Urumqi, 830054 China; 4 China National Agro-Tech Extension and Service Center, Beijing, 100125 China; 5 Songjiang District Agro-Technology Extension Center, Shanghai, 201613 China; 6 Natural Resources Institute, University of Greenwich, Chatham, ME4 4TB UK; 7 Rothamsted Research, Harpenden, AL5 2JQ UK; 8 Centre of Ecology and Conservation, and Environment and Sustainability Institute, University of Exeter, Penryn, Cornwall, TR10 9FE UK

**Keywords:** Insect migration, East Asian monsoon, Wind pattern, Population dynamics, Lepidoptera

## Abstract

**Background:**

In warm regions or seasons of the year, the planetary boundary layer is occupied by a huge variety and quantity of insects, but the southward migration of insects (in East Asia) in autumn is still poorly understood.

**Methods:**

We collated daily catches of the oriental armyworm (*Mythimna separata*) moth from 20 searchlight traps from 2014 to 2017 in China. In order to explore the autumn migratory connectivity of *M. separata* in East China, we analyzed the autumn climate and simulated the autumn migration process of moths.

**Results:**

The results confirmed that northward moth migration in spring and summer under the East Asian monsoon system can bring rapid population growth. However, slow southerly wind (blowing towards the north) prevailed over the major summer breeding area in North China (33°–40° N) due to a cold high-pressure system located there, and this severely disrupts the autumn ‘return’ migration of this pest. Less than 8% of moths from the summer breeding area successfully migrated back to their winter-breeding region, resulting in a sharp decline of the population abundance in autumn. As northerly winds (blowing towards the south) predominate at the eastern periphery of a high-pressure system, the westward movement of the high-pressure system leads to more northerlies over North China, increasing the numbers of moths migrating southward successfully. Therefore, an outbreak year of *M. separata* larvae was associated with a more westward position of the high-pressure system during the previous autumn.

**Conclusion:**

These results indicate that the southward migration in autumn is crucial for sustaining pest populations of *M. separata*, and the position of the cold high-pressure system in September is a key environmental driver of the population size in the next year. This study indicates that the autumn migration of insects in East China is more complex than previously recognized, and that the meteorological conditions in autumn are an important driver of migratory insects’ seasonal and interannual population dynamics.

**Supplementary Information:**

The online version contains supplementary material available at 10.1186/s40462-022-00360-3.

## Background

Migration is an important part of the life history of many animals, and it is a successful survival strategy when confronted by spatial and temporal heterogeneity of natural resources [1]. Among the insects, many species use wind-assisted migration within the planetary boundary layer [2] and so, under warm conditions, this layer of the atmosphere may contain huge numbers of migrants [3]. Mass insect migration is an important long-distance transport process, with implications for predator–prey interactions, essential nutrient cycles in different ecosystems, pollination in natural and agricultural landscapes, and damage to agriculture and public health from pest insects [3–6]. In the Northern Hemisphere, many insects migrate northwards during spring and summer, and there is no doubt that some species can reap huge benefits by poleward movement into newly-available habitats at high latitudes. Benefits include increasing population abundance due to producing more generations per year, and leaving parasite- and natural enemy-infected habitats [7, 8]. The gradual deterioration of the environment during autumn requires many high-latitude migrants to move equatorward to avoid being stranded as winter approaches, and aspects of this return journey remain poorly known for most species.

Southward migration (in the Northern Hemisphere) in autumn was more rarely observed than northward migration, which puzzled early workers [9]. Some researchers believed seasonal poleward shifts to exploit temperate ecosystems represent a population sink from which progeny seldom return [10]. But more recently, increasingly more species have been confirmed to show return migrations in autumn [11–16]. For example, using the mark-release-recapture method, Showers et al. [16] proved that black cutworms (*Agrotis ipsilon*) in America can complete a southward migration of nearly 2000 km in autumn. Chapman et al. [7] estimated through insect trajectory analyses that 80% of the Silver Y moths (*Autographa gamma*) from the UK can reach warmer latitudes around the northern fringe of the Mediterranean, from approximately 50° N to 40° N. But several independent observations of different migratory insects have shown that the cumulative year-round observations of populations at low latitudes are significantly lower than those at mid-high latitudes [7, 17–19]. So, the question arises as to what's affecting the southward migration of insects in the autumn?

Weather systems with strong northerly winds (i.e., winds towards the south), such as cold fronts, provide a small ‘window’ for southward migration [16, 20, 21]. The seasonally reversed prevailing airflow in the monsoon climate zone [22] can also provide favorable airflow for the meridional migration of insects in autumn. However, due to the different temperature characteristics between maritime and monsoonal climates, migratory insects in East Asia require a lower latitude winter breeding area to survive in winter compared to Europe, and this may make their autumn migrations more challenging and riskier. In North China, spectacular migrations of many insect species have been observed over an island in the Bohai Strait in autumn, and the number of these autumn migrants was much greater than that of spring migrants [12–14]. However, most of these studies were from one site and did not assess how many migrating insects finally reached their destination, so the connectivity of autumn migration [23] and its implications for insect populations in East Asia remains unclear.

**Fig. 1:**
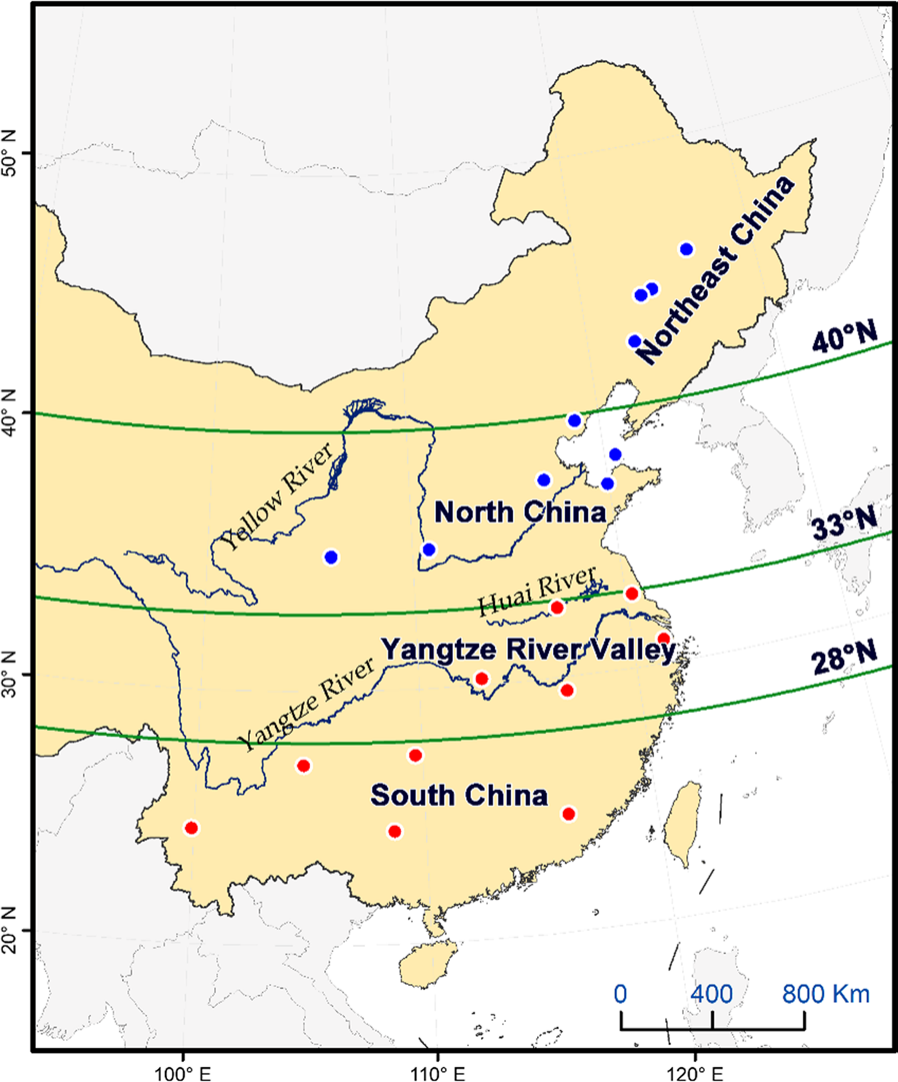
Map of the study area in China. Twenty searchlight traps were set up in 2014, ten of them were located in the overwintering area of *M. separata* (red points, to the south of 33° N), and other ten were located in the summer breeding area of this pest (blue points)

Here, we study the oriental armyworm (*Mythimna separata* Walker), one of the main agricultural pests that undertakes seasonal, long-distance, multigeneration roundtrip and meridional migration in East Asia [24, 25]. Its migration route in China has been clarified through many large-scale mark-release-recapture experiments in the 1960s and 1980s. Each spring, *M. separata* moths from their overwintering area (south of 0° C isotherm in January) migrate northward into North China, and further into Northeast China (Fig. 1). From mid to late July onwards, part of the *M. separata* populations from Northeast China have the opportunity to migrate south [24]. The autumn migration of this pest has been observed in August and September by a scanning radar located on an island in the Bohai Gulf, and migrating moths were moving to the southwest [12, 13, 26], but their autumn migration has not been studied at other places. *Mythimna separata* larvae are most destructive to crops in summer [27], but it is puzzling that larval outbreaks rarely occur in autumn in southern China, despite the huge numbers of the previous generation.

Currently, insect migrations over large spatial scales are hard to quantify [7, 28]. This study used widely deployed searchlight traps to compare the seasonal population dynamics of *M. separata* moths at different latitudes, with the aim of elucidating the role of autumn migration in the regulation of the annual migration cycles in this species (and perhaps other migrants in the region).

## Methods

### Monitoring migrant *M. separata* with searchlight trap data

To monitor the population dynamics of *M. separata*, the China National Agro-Tech Extension and Service Center has used 19 vertical-pointing searchlights as a monitoring network in different regions of the country since 2014 [29]; at the same time, we also set up a searchlight trap in Ningjin, Shandong province (Fig. 1). Based on years of understanding of the occurrence pattern of *M. separata* [24, 25, 27], searchlight traps were mainly placed along the migratory route and in the main outbreak areas of *M. separata*.

The vertical-pointing searchlight trap uses a 1000 W metal halide lamp, and it can effectively trap phototactic insects flying overhead up to about 500 m above, thus it can sample moths migrating at high altitude [13, 14]. Each trap was set in an open space without extensive maize-growing areas around it. These searchlight traps were run just in the major occurrence season of *M. separata* in 2014. Specifically, the searchlight traps in the Yangtze River Valley were run from February to May and from August to October, those in North China and Northeast China were run from May to September, and those in South China were run from September to next March. However, all searchlight traps were run all year round in the other three years (2015–2017). During the monitoring period, the searchlight traps were switched on each sunset (~ 19:00) and switched off the next dawn (~ 07:00), and catches were counted once a day.

**Fig. 2:**
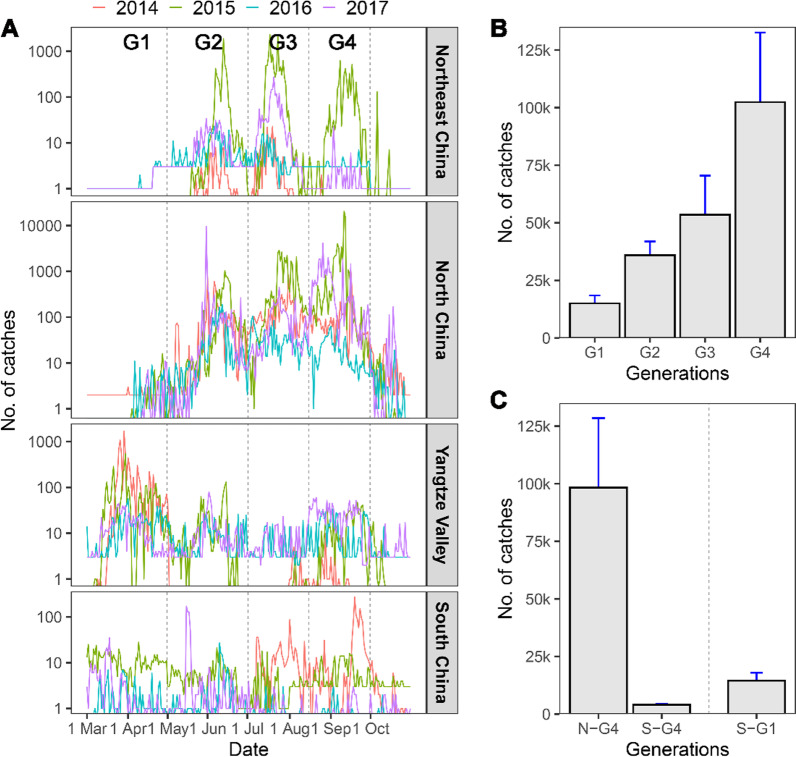
Seasonal population dynamics *M. separata* moths during 2014–2017. **A** Phenology of *M. separata* moths showing peaks that correspond to either migrants or emerged from local breeding. The whole migration circuit can be divided into four waves, and the fourth migration wave in the G4 period (from 16 August to 30 September) was the major southward migration as shown by increase of catches in the Yangtze Valley and South China. **B** The population increased through the first three migration waves. **C** The moth catches in the southern searchlight traps (to the south of 33° N) during G4 periods (S-G4) were much less than those in the northern searchlight traps (N-G4), and even less than those in the southern searchlight traps in spring (S-G1), indicating that most moths failed to reach overwintering areas during southward migration

The searchlight trap data of *M. separata* was split into four separate seasonal periods (Generation 1–Generation 4) based on our knowledge of migration patterns and local phenology [24, 25, 27] (Fig. 2). Generation 1 (G1) period was defined from 1 March to 30 April, G2 was from 1 May to 30 June, G3 period was from 1 July to 15 August, and G4 was from 16 August to 30 September.

### Occurrence levels of *M. separata* larvae

The historical data on the annual occurrence areas of *M. separata* larvae from 1959 to 2018 were obtained from the China National Agro-Tech Extension and Service Center [27, 30]. Based on this occurrence area dataset and knowledge from previous studies by Jiang et al. [27] and Liu et al. [30], the occurrence of *M. separata* larvae were classified into three levels: ‘outbreak’, ‘normal’ and ‘light’.

Outbreak years were defined as follows: (1) Before 1970, *M. separata* larvae outbroke frequently, but statistical omissions may have occurred due to technical reasons. Years in which the occurrence area was equal to or greater than that in 1960 were regarded as severe outbreak years, including 1960, 1966, and 1967; (2) Years (after 1970) with an occurrence area equal to or greater than that in 1987, including 1972, 1976, 1977, 1987, 1990, 2012 and 2013; (3) The occurrence degree of *M. separata* larvae during the 1990s was relatively light, but the occurrence area in 1998 was significantly higher than in other years, only slightly lower than in 1987.

The occurrence level of this pest in twenty-two years with the smallest occurrence area was defined as ‘light’, including 1961–1965, 1968–1969, 1988, 1992–1996, 2001, 2002, 2009–2011, and 2016–2018. The rest of the years were termed ‘normal’ years.

### Meteorological reanalysis data and climate conditions in autumn

In this study, we focused on the southward migration of *M. separata* moths in autumn. After late August, the summer populations of *M. separata* moths emerged in large numbers [25], and most of the moths were observed migrating at the height of 600–800 m in previous radar observations [14]. In general, most moths migrating at night move in approximately the same direction as the wind [31], so the wind direction in autumn will greatly affect the southward migration of mid to high latitude migrants. Therefore, the wind pattern at the 925 hPa level (about 800 m above sea level) from 16 August to 30 September in East China was explored. Examination of atmospheric circulation features during this period revealed that a cold high-pressure system at the 925 hPa level over North China was the major factor influencing the wind pattern.

In pursuit of higher resolution data, we used the ERA-interim (2002–2017) and ERA-40 (1959–2001) sets from the ECMWF (European Centre for Medium-Range Weather Forecasts) reanalysis data, with a resolution of 0.125° × 0.125°. These autumn climate analyses used the Monthly Means of Daily Means data under the ERA-interim data set, and the wind direction analysis was based on the daily data. All these data were downloaded from the Copernicus Climate Change Service (C3S) Climate Data Store (https://cds.climate.copernicus.eu).

Generally, northerly winds (blowing towards the south) mostly appear on the east side of cold high-pressure areas in the northern hemisphere, and thus the location of the cold high-pressure center (CPH) would influence the wind pattern greatly. We used the Grid Analysis and Display System (GrADS, version 2.0.1, http://cola.gmu.edu/grads/) to extract the coordinates of the maximum air pressure in the region of 115° E–125° E and 30° N–38° N at the 925 hPa height field in September each year. Two special cases indicate that the CHP may be outside the latitudes and longitudes defined above, and we need to expand the area appropriately: (1) If the longitude of the maximum pressure is at 115° E, set the range of the current year to 110° E–125° E, 30° N–38° N; (2) If the latitude of maximum pressure is at 38° N, set the range to 115° E–125° E, 30° N–42° N. Only the CHP in the southeast of the designated areas has biological significance for the autumn insect migration.

### Migratory trajectory analyses

We used the HYSPLIT model (Hybrid Single-Particle Lagrangian Integrated Trajectory Model, https://www.arl.noaa.gov/hysplit) to simulate the autumn migration of moths. This model was developed to analyze the transport and diffusion of atmospheric pollutants but it has also been used to simulate the migration trajectories of insects such as fall armyworm (*Spodoptera frugiperda*) and mosquitoes in recent years [18, 32]. Its principle is to regard the insect as a particle that moves with the airflow.

The searchlight trap located in the major food crop growing areas in summer and with the highest catches of *M. separata* moths was selected as the starting point for insect trajectory analysis, namely, Luanxian, longitude 118° 44′ 24″ E, latitude 39° 45′ 00″ N, located in the summer maize area in North China. (A total of 27,970 moths were captured in 171 days here from 2014 to 2017). The searchlight trap at Ningjin (longitude 116° 48′ 00″ E, latitude 37° 38′ 24″ N, where a total of 477 moths were captured in 51 days from 2014 to 2017), located at the south edge of North China, was also selected for insect trajectory analysis. We regarded Luanxian and Ningjin as representative of the summer breeding area population and we ran trajectory simulations on days when at least one moth was captured.

Radar observations show that *M. separata* moths, migrating at night, moved in the downwind direction, and that most of their flight altitudes in autumn were below 500 m, and the flight duration was less than 10 h [14]. Some results from tethered flight mill studies indicated that the flight ability of *M. separata* moths decreases significantly on the 6th day after emergence [33]. So, in order to explore the potential of *M. separata* moths to return to the south within a reasonable time range, we set the parameters of trajectory simulation based on the above research. We set the start time of the trajectory simulation to the local hour after sunset (Beijing time, UTC + 8 h), the time of each flight to 10 h, the trajectory height to 500 m, and we simulated trajectories for five consecutive nights, using the endpoint of the previous trajectory as the start point of the second. The self-powered flight speed of the moths was not considered in the trajectory analyses.

### Estimation of 5-night success rate in autumn migration

The northern boundary of the overwintering area of *M. separata* is around 33° N (the 0° C isotherm of average January temperature) [25], and moths must return to south of this geographic line in autumn in order to survive winter temperatures. However, reaching this line may not be enough to maintain the population size because prolonged exposure to temperatures over 0° C may also be deadly. For example, after 21 days at a constant temperature of 5° C, the mortality rate of the pupae exceeds 98%, and the 35-day mortality rate at 10° C exceeds 96% [34]. To return to winter breeding areas where the population can be maintained or increased, the fifth-generation *M. separata* in southern China must continue to migrate southward in mid-November [29]. Lin and Cheng (1958) found the effective accumulated temperature of the adult stage to be 685.2 degree days above 9.6 ± 1.0° C and 111.0 degree days above 9.0 ± 0.8° C, respectively [35]. Based on the effective accumulated temperature, the fourth-generation moths need to return to areas south of the 16° C isotherms in September–November (around 30° N). We use the ability to cross these two geographic lines (33° N and 30° N) as the basis for judging whether the simulated migration was successful.

To express the success rate of the southward migration objectively, we estimated two success rates based on the results of trajectory simulation. (1) From the population point of view, we regard the daily captured moths as an emigrating population, and estimated the success rate of this population (i.e., the proportion of endpoints south of 33° N/30° N to the total endpoints from 2014 to 2017). This method may reflect the long-term trend of the success rate more accurately. (2) From the individual point of view, the number of daily catches in the searchlight trap varies greatly during the autumn migration, so each endpoint represents a different number of moths. We estimated the proportion of individuals that successfully returned back to South China in the total catch from the north; this method can reflect the size of the return population more exactly, and there may be large inter-annual differences.

### Data analysis

We performed a Gaussian fit to the total catches and their latitudes at northern searchlight traps while describing the main summer breeding areas of armyworm. To test whether the longitude or latitude of the CHP in September affects the interannual variation in *M. separata* population abundance, we used the *one-sample t-test* (both longitude and latitude conform to normal distributions) and *t-test* for Pearson’s correlation coefficient. All Gaussian fit, t-test and correlation analyses were carried out in R (version 3.6, https://www.r-project.org/).

## Results

### Population increased along with northward migration

Based on the seasonal characteristics of the searchlight trap catches of *M. separata* moths in different regions from 2014 to 2017, the seasonal movement in China can be characterized by four waves of migration (Fig. 2A). In the first and second waves, *M. separata* moths migrated northward and expanded the distribution of the species from its overwintering areas (to the south of 33° N) in South China and the Yangtze River Valley into North China and even Northeast China. Then, the third migration wave occurred between Northeast China and North China in July and August. In the fourth migration wave (from 16 August onwards), moths migrated southward, as shown by the increased catches in the Yangtze Valley and South China (Fig. 2A).

During these four migration waves, the moth catches increased from generation to generation (Fig. 2B, Additional file 1: Table S1). Compared with the population in March and April (G1), the population size of G4 (from 16 August to 30 September) increased by 6.8 times on average in 2014–2017, with a growth rate (G4/G1) of 23.22 times in 2017, but only 0.72 times in 2014 (Additional file 1: Table S1). This result indicated that the population of migratory *M. separata* has huge annual fluctuations.

### Most moths failed to reach overwintering areas during southward migration

The major southward migration of *M. separata* moths during the G4 period (16 August-30 September) was the focus of this study. In total, 4060 moths were caught in the overwintering area (i.e., south of 33° N) in 2014–2017, but this was only 4.13% of the 98,293 moths caught to the north of 33° N (Fig. 2C). Moreover, *M. separata* adults were present to the south of 33° N all year round (Fig. 2A), and thus part of the catches here might be from the local population, not from migrants from the north; this was especially the case in years where the population size in the G4 period was quite small (Additional file 1: Table S2). For example, the ratio of catches between south and north of 33° N was up to 59.9% in 2016, but only 1026 moths were caught to the north of 33° N in this year. Therefore, it is inferred that mortality is high during the southward migration period, and a comparatively small proportion reached their overwintering area successfully. Moreover, the moth catches to the south of 33° N in the G4 period were even fewer than that in the G1 period (Fig. 2C, Additional file 1: Table S2), and this suggests that: (1) the large losses of moths during southward migration counteracted the population increase during the spring and summer migration period, and (2) the population must recover in size in its winter-breeding area.

### Wind pattern in September is often unsuitable for the southward migration of *M. separata* moths

**Fig. 3:**
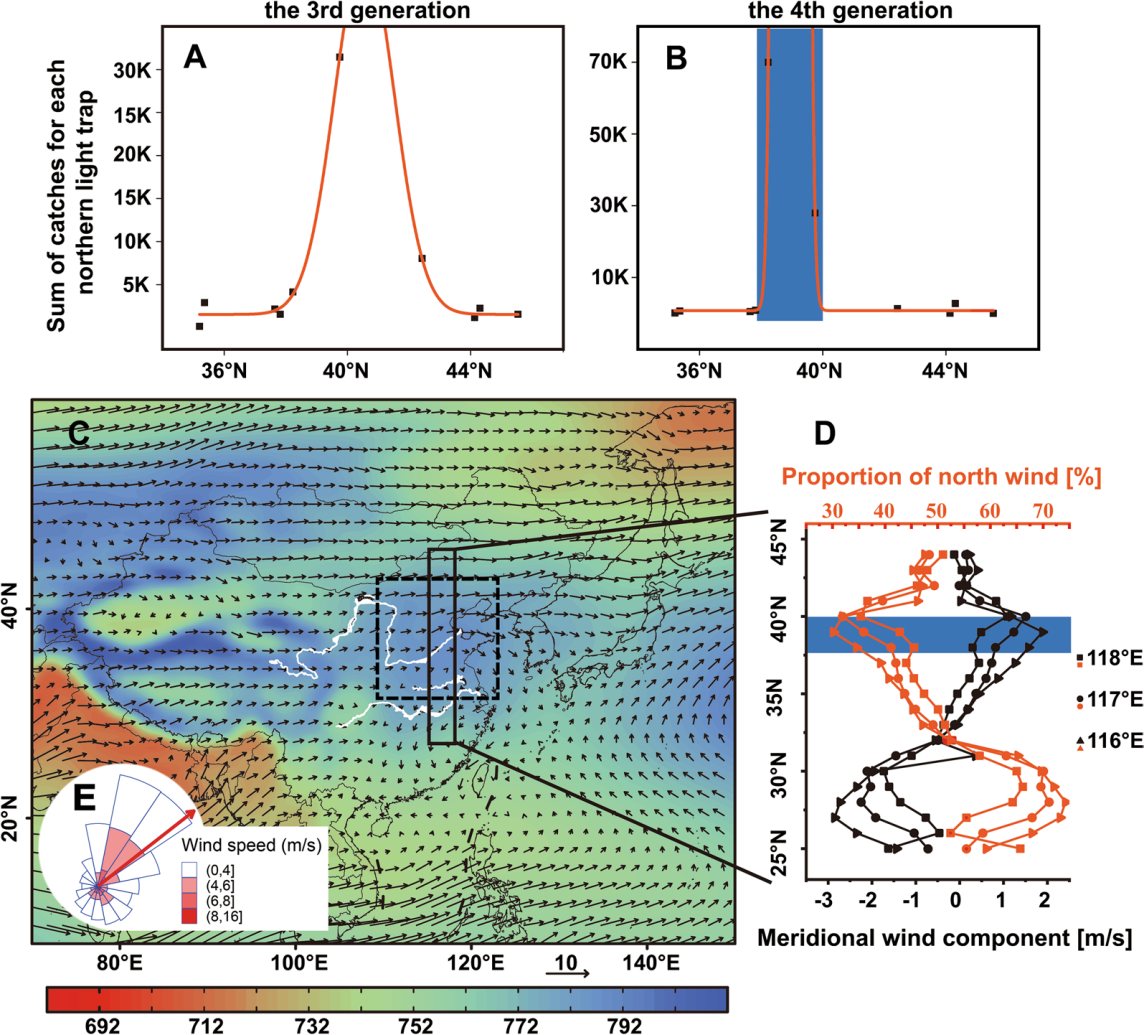
The wind pattern in North China is not suitable for the southward migration of *M. separata* moths in autumn. **A** **& B** The Gaussian fitting of the cumulative catches for each northern trap of the third (G3) and fourth (G4) generation of *M. separata* moths and the latitude of traps (G3: n = 10, F = 303.45, *p* = 0.000, r^2^ = 0.990; G4: n = 10, F = 1547.61, *p* = 0.000, r^2^ = 0.998). **C** The mean height field on 925 hPa in September from 1959 to 2017 showed that a high-pressure system covered North China (the area inside the dotted line). **D** Meridian wind in September in eastern China (116° E–118° E, mean for 1959–2017): the red line reflects the proportion of northerly winds at each latitude, and the black line reflects the meridional wind speed at each latitude (positive values indicate a south wind, negative values indicate a north wind). **E** A circular histogram showing the distribution of downwind direction and wind speed in North China in September

As mentioned previously, the major southward migration of *M. separata* moths occurred from 16 August to 30 September (Fig. 2A). To locate the latitude range of the source area of this migration wave, the variation of population size along latitudes was checked. Both the largest population size of *M. separata* in the G3 and G4 periods (1 July–30 September) were mostly near 39° N (Fig. 3A, B) and located in the summer maize area in North China. Here, the moth catches in the G3 period were immigrants from further afield and/or the locally-produced progeny of the G2 period. Thus, we infer that the large numbers of moths in North China mean that this is the major breeding area for G4 individuals, that the migrants during G4 periods are mostly from North China, but that most individuals migrate unsuccessfully over a short distance.

The weather conditions during the southward migration in the G4 period was explored. In late-August, the East Asian summer monsoon gradually weakens and moves southward, and at the same time, the East Asian winter monsoon began to affect North China. From the 925 hPa average height field in September, there was a high-pressure system over North China (Fig. 3C). High-pressure systems are usually associated with clear skies and calm weather, and thus the wind speed at 925 hPa over North China was quite slow (mean wind speed: 3.68 ± 0.03 m/s, n = 3540) and therefore not very suitable for the long-distance windborne transport of insects. From north to south in East China, the probability of experiencing a north wind fluctuated, first falling, then rising and falling again (see Fig. 3D); the minimum value appears around latitude 40° N, where the probability of a north wind is less than 40% (Fig. 3D). The wind direction in North China was mostly towards the northeast (Rayleigh test: n = 3540 mean direction = 51.92°, r = 0.345, *p* < 0.0001) (Fig. 3E). Therefore, most *M. separata* moths faced unfavourable airflow conditions during September’s southward migration.

### Modelling the success rate of *M. separata* moths during southward migration

**Fig. 4:**
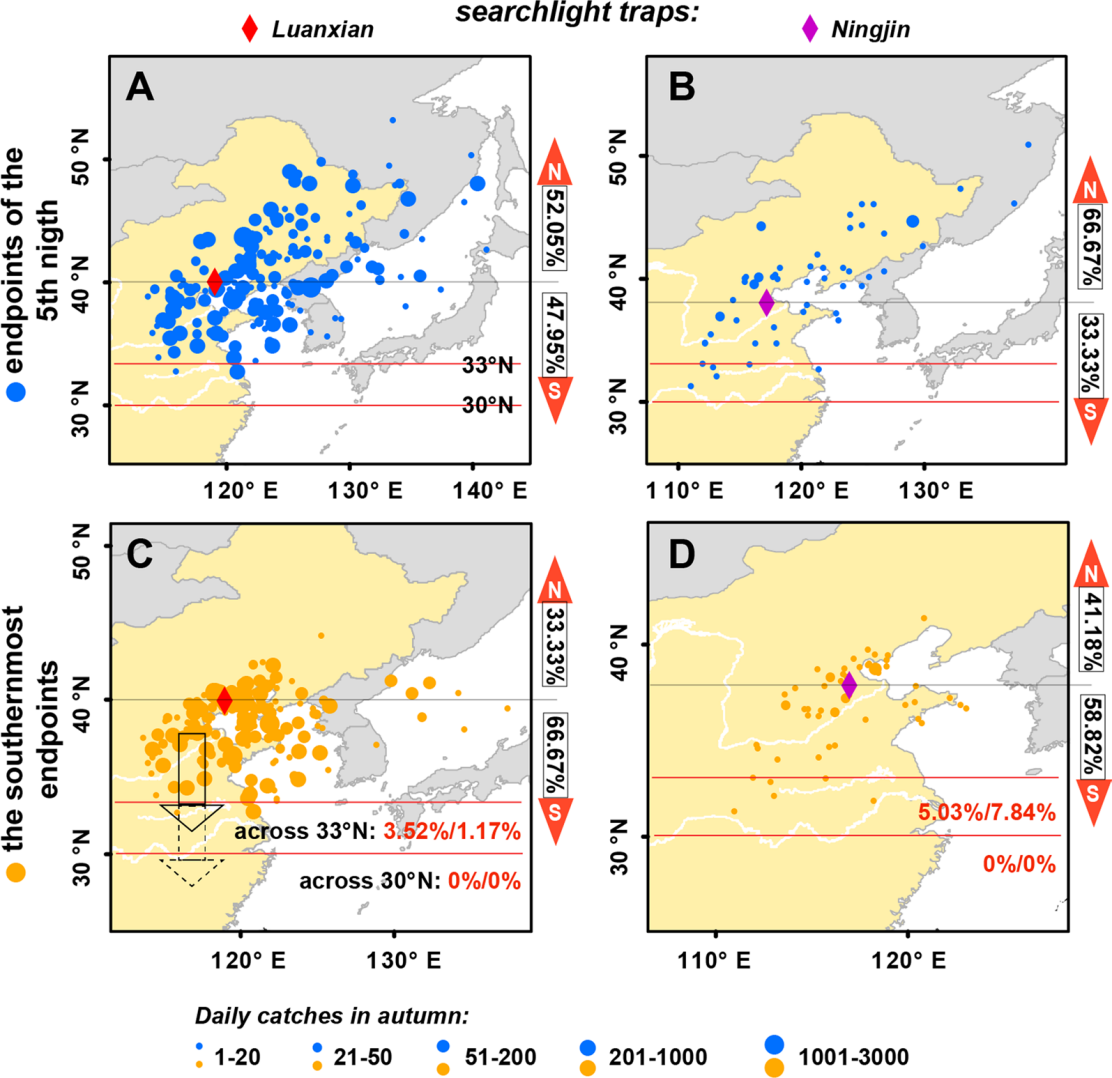
Daily trajectory simulations for the southward migration of the northern population of *M. separata* at Luanxian (LX) and Ningjin (NJ) during G4 periods (16 August–30 September) from 2014 to 2017. Each solid circle represents the whereabouts of a migrating population and contains two pieces of information: the size of the emigrating population and the endpoint of the migrating trajectory. After five nights of trajectory simulation, **A**, **B** Most simulated trajectories (LX: 52.05%; NJ: 66.67%) originating from North China finally moved towards the north. **C**, **D** Most of these simulated trajectories (LX: 66.67%; NJ: 58.82%) had southernmost endpoints located to the south of their origins, and a few of them (LX: 3.52%; NJ: 5.03%) reached south of latitude 33° N, but none of them reached south of 30° N. Taking the size of emigration populations as a weighting factor, the success rates of the southward migration from LX and NJ were 1.17% and 7.84%, respectively

To analyze the effect of autumn prevailing winds on migration, we quantified the southward migration success rates of *M. separata* in the G4 period using a trajectory analytical approach, with the Luanxian and Ningjin monitoring sites as the origin of the trajectories (Fig. 4). During the G4 period in 2014–2017, there were 171 nights in Luanxian and 51 nights in Ningjin when at least one moth was caught by searchlight traps, and thus in total 222 trajectories were calculated. After five days of migration, 55.4% (123/222) out of these trajectories eventually moved northward (Fig. 4A, B). This suggests that a large proportion of *M. separata* populations from North China were moving in an unfavourable direction during the G4 period.

The southernmost endpoints of the trajectories indicated the greatest potential for southward migratory distance, and we hypothesized that moths actively stopped migrating when these endpoint locations were reached. Among these 222 trajectories, 64.9% (144/222) of their southernmost endpoints were located to the south of their origins (Luanxian and Ningjin), and the distance of these southernmost endpoints from their origins were about 2.25° of latitude on average, and no more than 7° of latitude in maximum (Fig. 4C, D). Finally, only 6 trajectories (2.70%) reached the overwintering area (south of 33° N), and none of them reached south of 30° N where moths can breed in winter (Fig. 4C, D). If the daily catches are taken as an indication of emigrant population size and used as a weighting factor, the success rates of *M. separata* populations returning to the overwintering area were 3.52% and 5.03% in Luanxian and Ningjin, respectively. Not only that, the success rates of return migration estimated from trajectory simulations are close to those from the catches at searchlight traps (Figs. 2, 4). This agreement of the two results, i.e., trajectory simulations and the trap catches, confirms that migrating *M. separata* moths from North China in the G4 period failed to reach their overwintering area due to unsuitable wind patterns.

### Influence of the high-pressure system over North China on interannual variation of *M. separata* population abundance

**Fig. 5:**
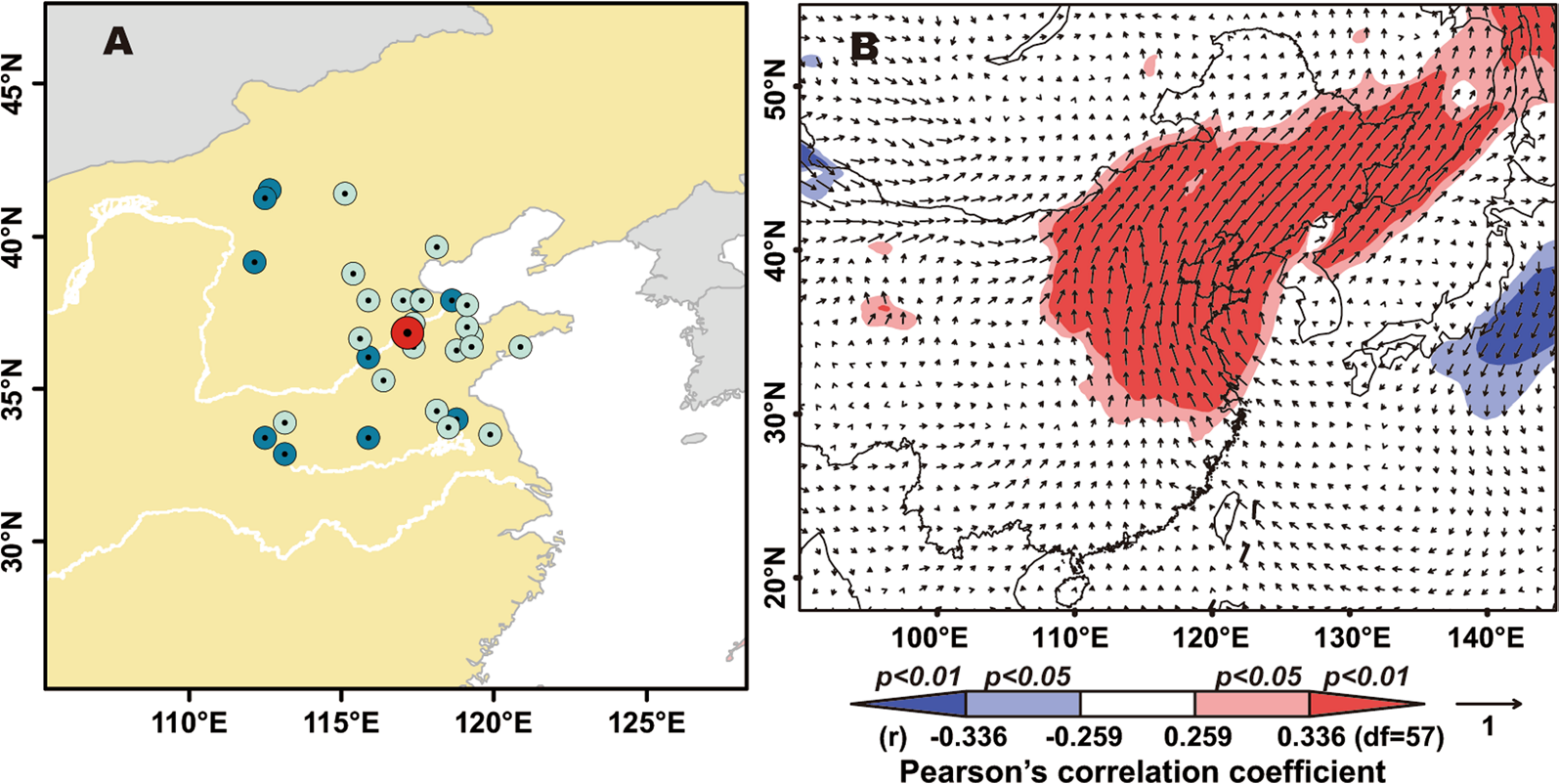
The location of the center of the high-pressure system (CHP) at 925 hPa drives the interannual variation in *M. separata* population dynamics. **A** The CHPs in the previous September of the year when armyworm larval outbreaks occurred (dark blue solid circles) mostly were located to the west of the mean position of CHPs in September from 1959 to 2017 (red solid circle). On the contrary, most CHPs in the previous September of the year with a light occurrence level of *M. separata* (light colored solid circle) were located to the east of the mean CHP. **B** Distribution of the Pearson’s correlation coefficients between the longitude of the CHPs and u/v component of winds at 925 hPa in September during 1959–2017. Dark and light shadings indicate regions where the Pearson’s correlation coefficients of the meridional component of wind pass the *t*-test at 0.01 and 0.05 levels, respectively. The blue area represents the significantly negative correlative region, while the red one represents the significantly positive correlative region

Most *M. separata* moths cannot migrate southward successfully to reach their overwintering area under the influence of the high-pressure weather system over North China. Therefore, we speculated that the southward migration in the G4 period is crucial for the *M. separata* population, and its annual population fluctuation would be driven by the characteristics of this high-pressure system. The centers of the high-pressure system (CHP) at 925 hPa from 1959 to 2017 were defined and extracted, and their mean coordinate in September was 117.17° E, 36.80° N. In the eleven years with an outbreak population of *M. separata*, the mean longitude of CHPs in the previous September was 115.18 ± 2.66° E, but it was at 117.54 ± 1.82° E for the 22 years when the occurrence level of this pest was light. The longitude values of CHPs between these two types of years were significantly different (Two-sample *t*-test: t = 2.647, df = 14.82, *p* = 0.018). In other words, CHPs in the previous September of the ‘outbreak’ years were located to the west of the mean CHP, and vice versa (Fig. 5A). Generally, a northerly wind (blowing towards the south) predominates at the eastern periphery of a high-pressure system, and thus the intensity of southerly winds (toward the north) over North and Northeast China was significantly correlated with the longitude of CHPs (Fig. 5B). Therefore, a westward position of the CHP results in more insects migrating southward successfully. Following from the above results, we suggest that the longitude of the CHP in September is a key factor determining the success rate of insect migration in autumn, and further influences the population size of northward migrants in the next spring.

## Discussion

Populations of *M.* separata emerging in late-summer or autumn at high latitudes need to migrate a linear distance of at least 600 km to return to the south of 33° N (Fig. 3B), which is undoubtedly a considerable challenge. Migratory insects have evolved a range of behavioral and physiological strategies to maximize their chances of successfully completing long-range movements to more favorable climes as local conditions deteriorate [28]. For example, numerous migratory insects select favorably-directed tailwinds for southward autumn migrations [3, 6, 7, 28], and newly-emerged rice leaf roller moths (*Cnaphalocrocis medinalis*), a major pest of rice in southern China, respond to deteriorating conditions such as food shortage by increasing their likelihood of migration in the next few nights compared to well-fed adult moths [36]. By contrast, however, *M. separata* moths respond to low temperatures and starvation within the first 24 h post-eclosion by shortening their pre-oviposition period (i.e., effectively reducing their window for migration, as the period of greatest flight activity is restricted to the pre-oviposition period) and increasing their fecundity [37]. This suggests that when autumn emigrant *M. separata* are carried in the wrong direction by prevailing winds, they may terminate their migration and bet-hedge against the risk of harsh environments by increasing the level of reproductive investment. This kind of strategy may explain why so few of the autumn generation of *M. separata* emigrants reach the permanent winter-breeding region.

Long-range migration of insects and birds is generally considered to be a costly strategy, with a high risk of mortality, especially during autumn migrations when conditions (weather, habitat suitability, food availability) tend to be deteriorating [38–45]. Severe weather does at times impact the migration of insects [2], and is also a major cause of mortality during bird migration [39], but such isolated and comparatively short-lived events are unlikely to explain seasonal and interannual population dynamics of insects such as *M. separata*. Extensive ecological barriers that must be crossed, such as deserts and seas, can also be a major mortality factor, especially for birds [41, 43]. However, this is unlikely to be an issue in East China as there are no large water bodies or deserts to cross. Food shortages along the migratory route, in the guise of availability of nectar plants, are important drivers of migration-related mortality in monarch butterflies (*Danaus plexippus*) [44, 45] and perhaps other migratory insects. The interannual population dynamics of *M. separata* in China are affected by the area of nectar sources along the migration route in spring [46], but the relationship between availability of nectar sources and *M. separata* migration mortality during autumn is unknown, and worthy of further study. But the most important driver of mortality and uncompleted autumn ‘return’ migrations in flying animals is likely to be the availability of suitable transport opportunities on winds blowing towards the south. Periods of unfavorable winds are known to affect mortality of migrant songbirds [47], but the impact will be even greater for insects given their comparatively slower speeds compared to typical winds. Previous studies have suggested that unfavorable autumn winds will hinder southward migrations of *M. separata* [26, 48], and our study provides strong support for this idea.

Southward autumn migrations are challenging for all insects, relying as they do on favorable tailwinds being frequent enough during the relatively short migration windows of insect migrants to enable several days or nights of transport in the seasonally appropriate direction. Some migratory insects achieve successful southward migration by the mechanisms of: (1) restricting migration to occasions with favorable winds, (2) selecting flight altitudes coincident with the optimal wind conditions, and (3) taking up headings that partially correct for drift while maximizing speed of transport [3, 6, 7, 28, 49]. Radar studies in East China indicate that *M. separata* moths tend to orientate downwind [14, 50], and our results indicate that favorably directed winds in the autumn are too scarce to allow many moths to reach the southern winter-breeding regions. In migratory species with longer adult lifespans and migration windows, such as birds [51, 52] and a few long-lived insects such as monarch butterflies and common green darner dragonflies (*Anax junius*) [53, 54], stopping over until winds and other weather conditions improve is a common strategy. This is unlikely to be feasible for migrant moths such as *M. separata*, as they have a limited window of opportunity in which to migrate—typically just the 4–5 nights immediately following adult eclosion [33]. Thus, in situations where winds are not frequently favorable for southward autumn migration, as in our study in East China, migratory moths presumably have to ‘make the best of a bad job’ and often fly on unfavorable winds, leading to the low success rates we observed.

Our results therefore indicate that during the autumn migration period the population undergoes an annual crash, as most moths will never reach a location suitable for producing the next generation. The small proportion of the migrants that reach the winter-breeding region are clearly crucial to the persistence of the population, and consequently the winter generations are the primary drivers of population growth in this species. This situation makes an interesting comparison to migratory insects that have been studied in Western Europe, where many insects have been observed to carry out successful mass migrations back to Mediterranean breeding grounds [3, 6, 7, 17, 28]. In this case, mortality along the autumn migration route is predicted to be comparatively small [7], and the populations appear to go through a ‘bottleneck’ during the winter in most (typical) years [7, 19]. This is because the winter-breeding regions (Maghreb and Sahel regions of north-west Africa) are usually rather arid, which restricts the growth of larval host plants [7], and it is only in atypically wet winters that large winter populations emigrate into Europe in the spring [19, 55]. Winter-breeding regions in South China by contrast do not go through such an extreme dry period, and as such the combination of moist, warm conditions provide perfect opportunities for population growth over the winter.

Given the relatively small proportion of the summer generations that return to the south, it is legitimate to pose the question as to why these moths migrate at all. We consider that seasonal changes in temperature drive the migration. Survival over the summer in South China will be severely compromised due to the climate in this region, as the eggs, larvae, and pupae of *M. separata* cannot survive temperatures > 35° C [56], and persistent temperatures > 30° C significantly reduce their reproductive abilities [57]. Thus, it is advantageous to depart the southern part of the range during the spring to escape the adversely hot temperatures. Conversely, autumn temperatures in North China rapidly drop, and this will cause the population to crash as the developmental period of *M. separata* is extended by nearly 100 days when the temperature drops from 20 to 15° C [35]. Thus, a rapid retreat to the south will be highly beneficial for those migrants that can make it. Another benefit that will accrue to migrants relative to individuals that remain in the same area year-round is migratory escape to comparatively enemy-free spaces, and consequently lower rates of parasitism and disease [8, 28]. These benefits presumably favored the evolution of migration in *M. separata* despite the risks and high levels of mortality associated with the migratory journey. Winter areas not only play a decisive role in regulating summer population abundance [19, 55] but also play an important role in regulating migratory populations (Fig. 2C) [58]. A long-term stable winter breeding area is extremely important for any seasonal migration pattern, and considering that migratory behaviors are currently threatened to varying degrees around the world [9], the protection of winter-breeding areas of non-pest species will be a key part of human intervention [59]. In the management of migratory pests, it is also essential to carry out comprehensive research in the source regions. We need to raise the focus on winter-breeding areas to a higher level, and these need to be based on more comprehensive and in-depth cross-border cooperation.

## Conclusions

The influence of cold high-pressure systems on autumn wind patterns in East China seems to control the success rate of the southward ‘return’ migration of *M. separata* moths which have bred in North and Northeast China. A greater proportion of moths successfully returned to the south in years when the center of the high-pressure system (CHP) in September was further to the west than typical, and this increased the possibility of larval outbreaks in the next year [60]. Our results indicate that the southward migration in autumn is crucial for sustaining pest-level populations of this species, and the September position of the CHP is a key environmental driver of this process. Our study reveals factors influencing seasonal population fluctuations of a long-distance migratory insect pest, and provides crucial information for predicting outbreak populations.

## Supplementary Information

**Additional file 1: Table S1**. Cumulative light-trap catches of *M. separata* in each generation in 2014–2017. **Table S2**. Cumulative light-trap catches of *M. separata* in north and south regions.

## Data Availability

Excel spreadsheets of population data have been deposited in Dryad, https://doi.org/10.5061/dryad.0vt4b8h38 [61].
