# Supplementary material for: A cold high-pressure system over North China hinders the southward migration of Mythimna separata in autumn
Source: Mov Ecol. 2022 Dec 1;10:54. doi: 10.1186/s40462-022-00360-3 (PMC9716675; doi:10.1186/s40462-022-00360-3)
Supplement: Supplementary file 1 — Additional file 1: Table S1. Cumulative light-trap catches of M. separata in each generation in 2014–2017. Table S2. Cumulative light-trap catches of M. separata in north and south regions. [file 40462_2022_360_MOESM1_ESM.docx]

Additional file 1: Table S1. Cumulative light-trap catches of *M. separata* in each generation in 2014–2017

| Year | G1  (Mar-Apr) | G2  (May-Jun) | G3  (1 Jul-15 Aug) | G4  (16 Aug-30 Sept) | G4/G1 |
| --- | --- | --- | --- | --- | --- |
| 2014 | 8391 | 4841 | 7027 | 6009 | 0.72 |
| 2015 | 4316 | 13352 | 38632 | 67793 | 15.71 |
| 2016 | 1159 | 2908 | 2073 | 1641 | 1.42 |
| 2017 | 1159 | 14827 | 5739 | 26910 | 23.22 |
| Total | 15025 | 35928 | 53471 | 102353 | 6.81 |

Additional file 1: Table S2. Cumulative light-trap catches of *M. separata* in north and south regions

| Year | N-G4  (North searchlight traps) | S-G4  (South searchlight traps) | S-G1 | S-G4/N-G4 | S-G4/G1 |
| --- | --- | --- | --- | --- | --- |
| 2014 | 4782 | 1227 | 8266 | 0.26 | 0.15 |
| 2015 | 66830 | 963 | 4246 | 0.01 | 0.23 |
| 2016 | 1026 | 615 | 993 | 0.60 | 0.62 |
| 2017 | 25655 | 1255 | 1023 | 0.05 | 1.23 |
| Total | 98293 | 4060 | 14528 | 0.04 | 0.28 |

Southern searchlight traps were located to the south of 33°N, while northern searchlight traps were located to the north of 33°N (see Figure 1).
